# Many human pharmaceuticals are weak inhibitors of the cytochrome P450 system in rainbow trout (*Oncorhynchus mykiss*) liver S9 fractions

**DOI:** 10.3389/ftox.2024.1406942

**Published:** 2024-07-15

**Authors:** Tea Pihlaja, Timo Oksanen, Netta Vinkvist, Tiina Sikanen

**Affiliations:** ^1^ Faculty of Pharmacy, Drug Research Program, University of Helsinki, Helsinki, Finland; ^2^ Helsinki Institute of Sustainability Science, University of Helsinki, Helsinki, Finland

**Keywords:** cytochrome P450, enzyme inhibition, pharmaceuticals, rainbow trout, ecotoxicology, bioaccumulation

## Abstract

**Introduction:**

Pharmaceutical residues are widely detected in aquatic environment and can be taken up by nontarget species such as fish. The cytochromes P450 (CYP) represent an important detoxification mechanism in fish, like in humans. In the present study, we assessed the correlation of the substrate selectivities of rainbow trout CYP1A and CYP3A homologues with those of human, through determination of the half-maximal inhibitory concentrations (IC_50_) of a total sixteen human pharmaceuticals toward CYP1A-like ethoxyresorufin O-deethylase (EROD) and CYP3A-like 7-benzyloxy-4-trifluoromethylcoumarin O-debenzylase (BFCOD) in rainbow trout (*Oncorhynchus mykiss*) liver S9 fractions (RT-S9).

**Methods:**

The inhibitory impacts (IC_50_) of atomoxetine, atorvastatin, azelastine, bimatoprost, clomethiazole, clozapine, desloratadine, disulfiram, esomeprazole, felbinac, flecainide, orphenadrine, prazosin, quetiapine, sulpiride, and zolmitriptan toward the EROD and BFCOD activities in RT-S9 were determined using the IC_50_ shift assay, capable of identifying time-dependent inhibitors (TDI). Additionally, the nonspecific binding of the test pharmaceuticals to RT-S9 was assessed using equilibrium dialysis.

**Results:**

Most test pharmaceuticals were moderate to weak inhibitors of both EROD and BFCOD activity in RT-S9, even if most are noninhibitors of human CYP1A or CYP3A. Only bimatoprost, clomethiazole, felbinac, sulpiride, and zolmitriptan did not inhibit either activity in RT-S9. EROD inhibition was generally stronger than that of BFCOD and some substances (atomoxetine, flecainide, and prazosin) inhibited selectively only EROD activity. The strongest EROD inhibition was detected with azelastine and esomeprazole (unbound IC_50_ of 3.8 ± 0.5 µM and 3.0 ± 0.8 µM, respectively). None of the test substances were TDIs of BFCOD, but esomeprazole was a TDI of EROD. Apart from clomethiazole and disulfiram, the nonspecific binding of the test pharmaceuticals to the RT-S9 was extensive (unbound fractions <0.5) and correlated well (*R* ^2^ = 0.7135) with their water-octanol distribution coefficients.

**Discussion:**

The results indicate that the P450 interactions in RT-S9 cannot be explicitly predicted based on human data, but the *in vitro* data reported herein can shed light on the substrate selectivity of rainbow trout CYP1A1 and CYP3A27 in comparison to their human homologues. The IC_50_ concentrations are however many orders of magnitude higher than average environmental concentrations of pharmaceuticals. The time-dependent EROD inhibition by esomeprazole could warrant further research to evaluate its possible interlinkages with hepatotoxic impacts on fish.

## 1 Introduction

Residues of active pharmaceutical ingredients, primarily arising from human excretions in the sewage, are widely detected in surface waters all around the world (Aus der Beek et al., 2016; Wilkinson et al., 2022). Currently, environmental risk assessment (ERA) is mandatory for the marketing authorization of new active pharmaceutical ingredients (APIs) in the European Union, the United States and Canada (Lee and Choi, 2019), but overall, there is very limited data on the environmental effects and fate of the thousands of “legacy” APIs in clinical use worldwide. At the same time, pharmaceuticals are in many ways a special class of environmental contaminants. Firstly, as a result of human use, their environmental exposure is continuous. They are also designed to be chemically stable in physiological conditions and are therefore not readily biodegradable, but often persistent in the environment. These characteristics increase the risk of pharmaceuticals’ accumulation in the environment over time. To facilitate their absorption and distribution across biological barriers, many pharmaceuticals are intrinsically relatively lipophilic. Furthermore, pharmaceuticals are designed to interact with specific human macromolecular protein targets at very low concentrations, to minimize their off-target effects in humans. As a consequence, the lipophilic pharmaceutical residues can be taken up by and cause a range of adverse effects in nontarget species. Owing to the high evolutionary conservation of human drug target proteins in fish (Gunnarsson et al., 2019), fish are among the most sensitive environmental organisms to the adverse effects of pharmaceutical residues. For example, steroid estrogen residues detected in European rivers have been associated with widespread sexual disruption (Jobling et al., 2006) and antipsychotic drug residues with behavioral changes (Brodin et al., 2013) in wild fish populations. Additionally, many antimicrobial, anti-inflammatory, and antipsychotic drugs have been reported to bioaccumulate in fish exposed to pharmaceutical mixtures in sewage effluents or treated wastewaters (Brown et al., 2007; Grabicova et al., 2017; Muir et al., 2017) as well as in wild fish (Cerveny et al., 2021).

How fish cope with pharmaceutical residues overall is largely determined by their chemical defensome, i.e., the integrative network of receptors and transcription factors, biotransformation enzymes, transporters, antioxidants, and metal- and heat-responsive genes, that collectively defend against and detoxify chemical stressors (Eide et al., 2021). These factors altogether determine, whether pharmaceutical residues are biotransformed and eliminated or if they bioaccumulate in fish tissues. According to current understanding, not many pharmaceuticals are inherently very bioaccumulative in fish, when assessed one at a time using standardized tests (Gómez-Regalado et al., 2023). For one part, many pharmaceuticals are ionizable substances, which can reduce their lipophilicity at environmental and physiological pH, and thus bioavailability in fish. For the other part, fish have similar enzyme systems as humans, that can biotransform the initially lipophilic active substances into readily excretable, typically more water-soluble and inactive metabolites. In fish, these enzymes are largely expressed in both hepatic and many extrahepatic (e.g., gill, intestine, brain) tissues and include primarily cytochromes P450 (CYP) and a range of conjugation enzyme systems, such as UDP-glucuronosyl transferases (UGT), sulfotransferases (SULT), and glutathione-S-transferases (GST) (Franco et al., 2024).

Biotransformation can however also result in formation of reactive and thus toxic metabolites. In humans, such metabolites are primarily formed via the oxido-reductive reactions catalyzed by the cytochrome P450 system (Guengerich, 2006). Owing to the relatively narrow substrate specificity of human P450s (compared with that of human UGTs, SULTs or GSTs) (Dong et al., 2012), pharmaceuticals binding to the P450 isoenzymes can also result in reversible or even irreversible enzyme inhibition. Irreversible P450 binding is the most severe form of inhibition because it results in a long-lasting (days) enzyme inactivation until *de novo* synthesis of new functional enzymes. It usually derives from activation of the parent pharmaceutical by P450s into a reactive metabolite, which tightly binds to the enzyme active site (Fontana et al., 2005). Subsequently, the covalent bond between the metabolite and the enzyme can lead to hapten formation, which can in some cases trigger an autoimmune response resulting in hepatotoxicity in humans (Li, 2002). Therefore, the risk for irreversible inhibition of human P450s is screened as early as possible in the drug discovery process, typically using an *in vitro* IC_50_ shift assay (Obach et al., 2007; Berry and Zhao, 2008), which can identify time-dependent inhibitors.

Previous literature has already established evidence of the fact that human pharmaceuticals can have similar inhibitory impacts toward selected fish P450 enzyme activities *in vitro* and *in vivo*, such as the CYP1A-like ethoxyresorufin O-deethylase (EROD) and the CYP3A-like 7-benzyloxy-4-trifluoromethylcoumarin O-debenzylase (BFCOD) (Miranda et al., 1998; Smith et al., 2012; Burkina et al., 2013; Pihlaja et al., 2022). Although these P450 activities are also known to be inducible in fish upon chemical exposure (Whyte et al., 2000; Oziolor et al., 2017; Burkina et al., 2021), the induction and *de novo* protein synthesis typically take several days, whereas the inhibitory effects are instant. Consequently, fish P450 inhibition by one or more pharmaceuticals can temporarily prevent the biotransformation of other substances and reduce their intrinsic clearances, if the overall detoxification is critically dependent on the inhibited P450 route. However, to be able to evaluate, if this mode of action is a true risk at environmental concentrations of pharmaceuticals (typically in the ng/L-µg/L range (Aus der Beek et al., 2016; Wilkinson et al., 2022)), it is critical to know the threshold inhibitory concentrations of pharmaceuticals toward the fish P450 system. In humans, the half-maximal inhibitory concentrations (IC_50_) of pharmaceuticals are typically in the micromolar range (equivalent to low mg/L range), i.e., many orders of magnitude higher than their average environmental concentrations or fish tissue concentrations (e.g., Grabicova et al., 2017). For fish, however, the threshold concentrations of P450 inhibition are not as thoroughly studied and the data for pharmaceuticals is particularly scarce.

The aim of the present study was to evaluate the propensity of fish P450 inhibition by human pharmaceuticals *in vitro* and its possible impact on xenobiotic elimination and bioaccumulation in fish exposed to pharmaceutical mixtures. For this purpose, we determined the threshold inhibitory concentrations (IC_50_) of a total of sixteen human pharmaceuticals (Table 1), from different therapeutic classes, toward the EROD and BFCOD activities in rainbow trout (*Oncorhynchus mykiss*) liver S9 fractions (RT-S9). Rainbow trout was chosen as the model organism, because it is one of the recommended fish species for exposure (OECD203) and bioaccumulation (OECD305) assessment owing to its sensitivity to chemical contaminants. Although extrahepatic metabolism has an essential role with respect to the total chemical defensome, liver has the highest activities for most enzyme systems that contribute to the biotransformation of pharmaceuticals in rainbow trout, including CYP1A and CYP3A (Franco et al., 2024). For one part, the objective of the study was to assess qualitative correlation of the substrate selectivities of rainbow trout CYP1A and CYP3A with those of their human homologues, to evaluate the possibilities for read-across from human P450 inhibition data. Another objective of the study was to evaluate the biological importance of P450 inhibition to pharmaceuticals’ bioaccumulation in rainbow trout based on the IC_50_ concentrations and through subsequent identification of possible time-dependent inhibitors via the IC_50_ shift, which could be indicative of the formation of reactive and thus toxic metabolites in fish liver.

**TABLE 1:** Overview of the test substances, their molecular properties and human cytochrome P450 (CYP) 1A and 3A interactions. (e) denotes empirical and (p) predicted (DrugBank, admetSAR). For human P450 interactions (m) denotes moderate inhibition with 1 µM < IC_50_ < 10 μM, (w) weak inhibition with IC_50_ > 10 μM, NS nonsubstrate, and NI noninhibitor.

Test substance	Therapeutic use^*^	MW (g/mol)	Water solubility ** (mg/mL, pH 7.8)	pK_a_ strongest acid/base^**^ (charge state at pH 7.8)	LogK_OW_^**^	LogD^**^ (pH 7.8)	Human CYP1A interactions^a^	Human CYP3A interactions^a^
**Atomoxetine** hydrochloride	hyperactivity disorder	291.82	27.8 (e)	9.4, base (positive)	3.809 (p)	2.20 (p)	NS, NI	NS, NI
**Atorvastatin,** calcium trihydrate	cholesterol lowering drug	1209.41	0.1525 (p)	4.31, acid (negative)	6.36 (e)	2.18 (p)	NS, NI	Substrate, Inhibitor (w)^1^
**Azelastine,** hydrochloride	antihistamine	418.36	0.1131 (p)	8.58, base (positive)	4.9 (e)	3.20 (p)	Substrate (p), NI	Substrate (p), Inhibitor (m)
**Bimatoprost**	glaucoma ocular hypertension	415.57	0.0527 (p)	14.35, weak acid (neutral)	3.2 (e)	2.63 (p)	NS, NI	Substrate, NI
**Clomethiazole,** hydrochloride	sedative, anticonvulsant	161.65	1.69 (p)	3.18, weak base (neutral)	2.1 (p)	1.77 (p)	NS, NI	Substrate, NI
**Clozapine**	schizophrenia, antipsychotic	326.83	0.114 (p)	8.16, base (positive/neutral)	3.23 (e)	2.88 (p)	Substrate^2^, NI	Substrate^2^, NI
**Desloratadine**	antihistamine	310.83	14.4 (p)	10.13, base (positive)	3.2 (e)	1.69 (p)	NS, Inhibitor (p)	NS (p), NI (p)
**Disulfiram**	chronic alcoholism, antabus	296.52	0.0014 (p)	neutral	3.88 (e)	4.16 (p)	NS, NI	NS, NI
**Esomeprazole,** magnesium hydrate	indigestion, heartburn acid reflux	345.42	0.109 (p)	9.68, acid (neutral)	2.2 (p)	2.43 (p)	NS, NI	Substrate, Inhibitor (p)
**Felbinac**	anti-inflammatory drug	212.25	80.6 (p)	4.73, acid (negative)	3.258 (p)	0.32 (p)	NS (p), NI	NS (p), NI
**Flecainide,** acetate	anti-arrhythmic drug	414.35	0.241 (p)	9.62, base (positive)	3.78 (e)	1.38 (p)	Substrate^3^, NI	NS, NI
**Orphenadrine,** citrate	skeletal muscle relaxant	461.51	12.2 (p)	8.87, base (positive)	3.77 (e)	3.06 (p)	Substrate (p), Inhibitor (w)^4^	Substrate (p), Inhibitor (w)^4^
**Prazosin,** hydrochloride	hypertension	418.86	0.0043 (p)	7.92, base (positive/neutral)	1.3 (p)	1.29 (p)	Substrate (p) NI (p)	Substrate (p) NI (p)
**Quetiapine,** hemifumarate	schizophrenia, bipolar disorder, antipsychotic	383.51	0.0397 (p)	7.76, base (positive/neutral)	2.81 (e)	2.53 (p)	NS, NI	Substrate, NI
**Sulpiride**	schizophrenia, antipsychotic	341.43	52.2 (p)	8.97, base (positive)	0.57 (e)	−0.88 (p)	NS, NI^5^	NS, NI^5^
**Zolmitriptan**	migraine	287.36	78.3 (p)	9.7, base (positive)	1.792 (e)	0.27 (p)	Substrate, NI	NS, NI

* Therapeutic use and human metabolism data collected from Drugbank (https://go.drugbank.com) unless otherwise stated. ** Water solubility and predicted LogK_OW_, and LogD_OW_ values are from Chemaxon (https://chemaxon.com/) and experimental LogK_OW_ from PubChem (https://pubchem.ncbi.nlm.nih.gov).

^1^ Cohen et al., 2000; ^2^ Dragovic et al., 2013; ^3^ Conard and Ober 1984; ^4^ Guo et al., 1997; ^5^ Niwa et al., 2005.

## 2 Materials and methods

### 2.1 Rainbow trout liver S9 fractions

Commercially available rainbow trout (*O. mykiss*) liver S9 fractions were used as the enzyme source for the *in vitro* P450 inhibition assays (RTL-S9, total protein concentration 20 mg/mL, Primacyt Cell Culture Technology GmbH). The RTL-S9 lot used (#RTL200629-3) was a mixed gender pool of seven fish (two male and five female; Strain: Christophersen, Bornhoeved; Supplier: Fish breeding Christophersen; Acclimation temperature: 8.8°C ± 3.4°C; Age: approx. 1.5–2 years; Fish weight 426 ± 31 g; Liver weight 4.0 ± 0.5 g; Gonadosomatic index: 0.03–0.36).

The RT-S9 is a subcellular fraction of rainbow trout liver cells, prepared via centrifugation (see, e.g., the OECD test guidance no. 319B). The S9 fractions comprise both microsomal enzymes, such as the P450s (phase I oxido-reductive reactions) and UGTs (phase II, glucuronide conjugation), and cytosolic phase II conjugation systems, such as SULTs and GSTs. The characterization data for these enzyme activities in the commercial RT-S9 lot used in this study, as provided by the supplier, are given in the Supplementary Table S1.

However, the S9 fraction does not readily contain endogenous cofactors, such as β-nicotinamide-adenindinucleotide-2-phosphate (NADPH, cofactor of P450s), UDP-glucuronic acid (UDPGA, cofactor of UGTs), 3′-phosphoadenosine-5′-phosphosulfate (PAPS, cofactor of SULTs), or glutathione (cofactor of GSTs), but these need to be separately added to the RT-S9 incubations to activate the respective enzyme systems. As a result, the subcellular RT-S9 fractions enable detailed mechanism-based studies via selective activation of dedicated clearance routes by the addition of the corresponding cofactor, such as NADPH to activate the P450 system only, or all cofactors to activate all aforementioned enzyme systems simultaneously.

### 2.2 Chemicals and reagents

Potassium phosphate buffer (0.1 M, pH 7.8) was prepared from dipotassium hydrogen phosphate (Amresco) and potassium dihydrogen phosphate (Riedel-de-Haën). The cofactors β-nicotinamide-adenindinucleotide-2-phosphate (NADPH, reduced tetrasodium salt hydrate, >93%), uridine 5′-diphosphoglucuronic acid (UDPGA, trisodium salt), L-Glutathione (GSH, reduced), and adenosine 3-phosphate 5-phosphosulfate (PAPS, lithium salt hydrate, >60%) were purchased from Sigma-Aldrich, and alamethicin from A.G. Scientific Inc.

The pre-fluorescent P450 model substrates, 7-ethoxyresorufin (ER) and 7-benzyloxy-4-trifluoromethyl coumarin (BFC), were purchased from Toronto Research Chemicals (TRC) and Apollo Scientific Ltd, respectively, and the corresponding metabolite standards resorufin and 7-hydroxy-4-trifluoromethyl coumarin (HFC) from Sigma Aldrich and TRC, respectively. The stock solutions of ER and BFC were prepared in dimethylsulfoxide (DMSO, Sigma Aldrich) and acetonitrile (Riedel-de-Haën), respectively, and diluted with the phosphate buffer before use, so that the residual solvent concentration resulting from the model substrate was constant in all incubations, either 0.5%, v/v, DMSO (EROD) or 2%, v/v, acetonitrile (BFCOD).

The test substances were purchased from Toronto Research Chemicals (bimatoprost, clomethiazole hydrochloride, disulfiram) or Sigma Aldrich (atomoxetine hydrochloride, atorvastatin calcium trihydrate, azelastine hydrochloride, clozapine, desloratadine, esomeprazole magnesium hydrate, felbinac, flecainide acetate, orphenadrine citrate, prazosin hydrochloride, quetiapine hemihumarate, sulpiride, zolmitriptan). The stock solutions of atorvastatin and prazosin were prepared in methanol (Riedel-de-Haën) and all other substances in DMSO. Before use, the stock solutions were diluted with the incubation buffer, so that the residual solvent concentration resulting from the pharmaceutical stock solution was constant (0.5%, v/v) in all incubations. All reagents and solvents used were of HPLC, LC-MS or analytical grade (≥98.0%) unless otherwise stated. Water was purified with a Milli-Q water purification system (Merck Millipore).

### 2.3 Determination of the half-maximal inhibitory concentrations of the test substances

The inhibitory impacts of the test substances toward the rainbow trout liver P450 system *in vitro* were assessed based on EROD and BFCOD as the marker reactions for CYP1A- and CYP3A-like isoenzyme activities, respectively. The enzyme-specificity of EROD and BFCOD in rainbow trout is well-established in previous literature (Hegelund et al., 2004; Jönsson et al., 2006; Christen et al., 2009; Burkina et al., 2021). In this study, the EROD (CYP1A) and BFCOD (CYP3A) activities were determined using 0.5 mg/mL RT-S9 (total protein) concentration and 1 or 2 mM NADPH, respectively. The concentrations of the marker substrates (1 μMER or 75 µM BFC) were adjusted close to their pre-determined K_M_ values (Pihlaja et al., 2022), and the incubation times pre-optimized (10 min for EROD and 20 min for BFCOD) to adhere to the Michaelis-Menten steady state assumption (i.e., linear metabolite formation rate over time). All incubations were performed in duplicate in a total volume of 100 µL of potassium phosphate buffer (0.1 M, pH 7.8), at 11°C ± 1°C on a thermostated orbital shaker (LLG Labware). The reactions were terminated by the addition of 37.5 μL of the quenching solution (0.5 M Trizma base/acetonitrile 20:80, v/v). To precipitate the proteins, the quenched reaction solutions were then kept on ice for at least 30 min and centrifuged (16,100 g, 15 min), after which the supernatants were analyzed for fluorescence to quantitate the marker metabolites, resorufin (ex/em 570/595 nm, EROD) and HFC (ex/em 419/501 nm, BFCOD). The quantitation of the metabolites (resorufin or HFC) was based on linear regression of the respective standards prepared in the potassium phosphate buffer. Negative control incubations were performed separately without the marker substrate, without cofactors, and without RT-S9 to ensure that the detected fluorescence signals arise from the metabolic conversion of the marker substrates (ER or BFC) into their respective metabolites (resorufin or HFC, respectively). Additionally, the impacts of the residual organic solvent concentrations on the basal EROD and BFCOD activities in rainbow trout *in vitro* was preliminarily determined to ensure that the marker reactions are not significantly inhibited by the organic solvent (see Supplementary Figure S1).

The half-maximal inhibitory concentrations (IC_50_) of the test substances were determined using the IC_50_ shift assay approach (Obach et al., 2007; Berry and Zhao, 2008), in which the test substance is first preincubated with RT-S9 for 30 min, without the marker substrate (ER or BFC). In this study, the preincubation was performed using three different protocols A-C (Table 2). In the first protocol, the test substance (each separately at six different concentrations between 0.01 and 500 µM) and RT-S9 were preincubated without any cofactors, and the marker reaction was initiated by the addition of the marker substrate (ER or BFC) and NADPH (Protocol A). This enabled the determination of the inhibitory impact of the parent form of the test substance on the respective marker activity. In the second protocol, the test substance and RT-S9 were preincubated together with NADPH, and the marker reaction was initiated by the addition of the marker substrate (Protocol B). In this manner, the test substance could undergo P450 metabolism already during the preincubation step, which may reduce its concentration and thus the inhibitory impact toward the marker reactions, or result in the formation of reactive or inhibitory metabolites, which typically increases the inhibitory impact compared with the parent form (Protocol A). In the third protocol, the test substance and RT-S9 were preincubated together with NADPH and the cofactors of the phase II enzyme systems, including UDPGA, PAPS, and glutathione, as well as alamethicin (25 μg/mL) (Protocol C). In this manner, the test substance could undergo both P450 and phase II metabolism already during the preincubation step, which may likewise reduce its concentration and inhibitory impact toward the marker reactions, and even help eliminate the possible reactive metabolites via glutathione conjugation. The cofactor concentrations in all incubations (Table 2) were adjusted well above the saturation levels to ensure that cofactor depletion during the preincubation step does not become the rate-limiting factor for the subsequent marker reaction. Additionally, positive control incubations of the marker substrates (ER or BFC) without the test substance (zero inhibitor concentration) were included in each series to determine the basal EROD or BFCOD activity in RT-S9.

**TABLE 2:** The marker substrate and cofactor concentrations used in the EROD and BFCOD inhibition assays for the different preincubation protocols A-C. The NADPH concentration in EROD (10 min) and BFCOD (20 min) reactions was 1 and 2 mM, respectively.

	Protocol A	Protocol B	Protocol C
**Preincubation with** 30 min	RTL-S9 (1 mg/mL) Test substance (0.01–500 µM)	RTL-S9 (1 mg/mL) Test substance (0.01–500 µM) + NADPH (1 or 2 mM)	RTL-S9 (1 mg/mL) Test substance (0.01–500 µM) + NADPH (1 or 2 mM) UDPGA (1 mM) PAPS (0.05 mM) Glutathione (2.5 mM)
**Marker reaction initiated with**	ER (1 µM) or (BFC 75 µM) + NADPH (1 or 2 mM)	ER (1 µM) or (BFC 75 µM)	ER (1 µM) or (BFC 75 µM)
**Endpoint**	Half-maximal inhibitory concentration (IC_50_) of the parent (API) form	Impact of test substance’s own P450 metabolism on its IC_50_ TDI if IC_50_ shift (A/B) ≥1.5	Impact of test substance’s own CYP and Phase II metabolism on its IC_50_

Finally, the EROD or BFCOD activities measured at different concentrations of the test substance were normalized to the corresponding basal activities (at zero concentration of the test substance) and the pharmaceuticals’ IC_50_ values were determined separately for each differently preincubated series using Graphpad Prism software version 9.4.1 (RRID:SCR_002798) and a nonlinear regression, without weighings (Eq. 1):

$$y=\frac{100}{1+10^{\left(LogIC50-x\right)\times HillSlope}}$$ (1)

where *y* is the relative activity (%) of the marker reaction compared with control incubation performed at zero pharmaceutical concentration, *x* is the concentration of the test pharmaceutical (µM), and Hill Slope is the steepness of the curve (constant value of −1). The residual solvent concentration was kept constant at all inhibitor concentrations, including zero, to eliminate its impact on the IC_50_ concentrations derived from the empirical data. The IC_50_ shifts for Protocols B and C were calculated according to Eq. 2:

$${IC}_{50\;}shift=\frac{{IC}_{50,Protocol\;A}}{{IC}_{50,Protocol\;B\;or\;Protocol\;C}}$$ (2)

In this manner, the impact of the test substance’s own metabolism (during the preincubation step) on the inhibitory impact could be evaluated. If the phase I (Protocol B) or phase I and II metabolism (Protocol C) results in signicant reduction in the concentration of the parent form during preincubation, the IC_50_ concentration increases compared with Protocol A, which yields an IC_50_ shift <1. However, if the metabolism of the parent form results in the formation of reactive and/or inhibitory metabolites, the IC_50_ decreases yielding IC_50_ shift >1 compared with Protocol A, with IC_50_ shift ≥1.5 as the commonly used threshold for time-dependent inhibition (Obach et al., 2007; Berry and Zhao, 2008).

### 2.4 Determination of the unbound fractions of the test substances in rainbow trout liver S9 fractions

The unbound (free) fractions of the test substances in RT-S9 (f_U,RT-S9_) were determined using rapid equilibrium dialysis assay (RED, Thermo Fisher Scientific), which exploits two-compartment inserts separated by a dialysis membrane with a molecular weight cut-off of 8,000 Da. In the RED assay, each test pharmaceutical (10 μM) was incubated in triplicate with RT-S9 (1 mg/mL) in 50 mM potassium phosphate buffer (pH 7.8) in a total volume of 300 µL on one side of the membrane (protein-containing chamber), and 350 μL of the plain phosphate buffer on the other side (protein-free chamber). To reach equilibrium, the samples were incubated on an orbital shaker (C25KC Incubator shaker, New Brunswick Scientific, 120 rpm) at 11°C for 4 h. Next, 50 µL aliquots were collected from both chambers separately and diluted by adding 50 µL of the plain buffer (to the protein-containing sample) or 50 μL of 1 mg/mL RT-S9 in the buffer (to the protein-free sample). Finally, the RT-S9 protein was precipitated by the addition of acetonitrile (+200 μL) to both samples separately, followed by incubation on ice for 30 min and centrifugation (16100 g, 15 min). The test substance concentrations in the supernatants of both samples were determined using liquid chromatography. The instrumentation and the analytical methods used for chromatographic analyses are described in detail in the Supplementary Table S2. The unbound fractions of the test substances in RT-S9 (f_U,RT-S9_) were calculated according to Eq. 3:

$$f_{U,RT-S9}=1-\;\frac{\left[PC\right]-\left[PF\right]}{\left[PC\right]}$$ (3)

where [PC] and [PF] are the test substance concentrations in the samples collected from protein-containing and protein-free chambers, respectively. To account for the test substance’s binding to the RT-S9, the nominal IC_50_ values were corrected for unbound fraction (f_U,RT-S9_) according to Eq. 4:

$${Unbound\;IC}_{50}={Nominal\;IC}_{50}\times f_{U,RT-S9}$$ (4)

## 3 Results

### 3.1 Inhibition of the EROD activity by the test substances in rainbow trout liver S9 fractions

**FIGURE 1:**
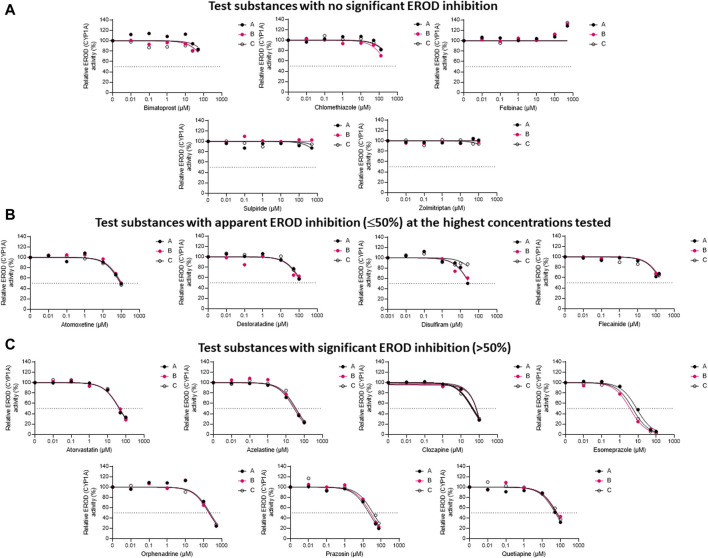
The nonlinear regression analyses of the residual EROD activities at different concentrations of the test pharmaceuticals in RT-S9, when the test substance and RT-S9 (1 mg/mL) were preincubated for 30 min without any cofactors (Series A), with 1 mM NADPH (Series B), or with all cofactors, including 1 mM NADPH, 1 mM UDPGA, 0.05 mM PAPS, and 2.5 mM glutathione (Series C), prior to initiation of the EROD marker reaction (10 min). On the basis of EROD inhibition, the test substances were grouped between **(A)** those that did not significantly inhibit EROD activity within the tested concentration range, **(B)** those that had apparent EROD inhibition of ≤50% at the highest test concentration, and **(C)** those that had >50% EROD inhibition at the highest test concentration. Each datapoint in each of the Series A-C represents an average of n = 2 replicate incubations.

The inhibitory impacts of the test substances toward rainbow trout CYP1A-like activity, assessed through nonlinear regression analyses of the residual EROD activities in RT-S9 assays at different pharmaceutical concentrations, are illustrated in Figure 1. On the basis of these results (Series A), five of the test substances (clomethiazole, bimatoprost, felbinac, sulpiride, and zolmitriptan) did not significantly inhibit the EROD activity in RT-S9 (Figure 1A). Apart from zolmitriptan (plausible human substrate), none of these substances have CYP1A interactions in human either (Table 1). Instead, all other test substances were shown to interact with CYP1A-like activity in RT-S9 and are categorized between those that caused weak, apparent inhibition (≤50%) at the highest concentrations tested (Figure 1B), and those that exhibited >50% inhibition and IC_50_ within the tested concentration range (Figure 1C). These substances included both substrates (azelastine, clozapine, flecainide) and inhibitors (desloratadine, orphenadrine) of human CYP1A, but most notably also a total of six substances that are neither substrates nor inhibitors of human CYP1A (atomoxetine, atorvastatin, disulfiram, esomeprazole, prazosin, quetiapine). Overall, the strongest EROD inhibition in RT-S9 was associated with esomeprazole (nominal IC_50_ = 8.4 ± 1.0 µM, Series A), followed by prazosin, azelastine, disulfiram, atorvastatin, clozapine and quetiapine, which all exhibited nominal IC_50_ values between 10 and 100 µM (Table 3). All other remaining substances (atomoxetine, desloratadine, flecainide, orphenadrine) exhibited only very weak inhibition with apparent IC_50_ > 100 µM.

**TABLE 3:** The nominal and unbound half maximal inhibitory concentrations (IC_50_) of the test substances toward EROD and BFCOD activities in RT-S9 following preincubation of the test substance only (Protocol A, bolded), as well as the IC_50_ shifts detected following preincubation of the test substance and RT-S9 with NADPH (Protocol B) or with all cofactors (Protocol C) prior to initiation of the marker reaction. (−) = no statistically significant inhibition. n/a = not applicable. The bold values refer to the unbound IC_50_ concentrations calculated according to Eq. 4, from the nominal IC_50_ of Protocol A.

Test substance	EROD inhibition in RT-S9			BFCOD inhibition in RT-S9			Unbound fraction (f_U,RT-S9_)	Range (µM)
	Nominal IC_50_ (µM)	IC_50_ shift	Unbound IC_50_ (µM)	Nominal IC_50_ (µM)	IC_50_ shift	Unbound IC_50_ (µM)		
**Atomoxetine (A)** *w/NADPH (B)* *w/cofactors (C)*	101 ± 12 110 ± 11 91 ± 8	0.9 ± 0.1 (A/B) 1.1 ± 0.2 (A/C)	**20 ± 4**	-	-	-	0.20 ± 0.03	0.01–100
**Atorvastatin (A)** *w/NADPH (B)* *w/cofactors (C)*	44 ± 8 46 ± 3 43 ± 5	1.0 ± 0.2 (A/B) 1.0 ± 0.2 (A/C)	**10 ± 2**	209 ± 46 *224 ± 32* -	0.9 ± 0.2 (A/B) n/a (A/C)	**45 ± 10**	0.22 ± 0.01	0.01–100
**Azelastine (A)** *w/NADPH (B)* *w/cofactors (C)*	28 ± 2 32 ± 4 36 ± 3	0.9 ± 0.1 (A/B) 0.8 ± 0.1 (A/C)	**3.8 ± 0.5**	237 ± 40 *324 ± 66* *432 ± 83*	0.7 ± 0.2 (A/B) 0.5 ± 0.1 (A/C)	**33 ± 6**	0.14 ± 0.01	0.01–100
**Clozapine (A)** *w/NADPH (B)* *w/cofactors (C)*	46 ± 9 45 ± 5 41 ± 11	1.0 ± 0.2 (A/B) 1.1 ± 0.4 (A/C)	**10 ± 3**	136 ± 8 *161 ± 15* *263 ± 71*	0.8 ± 0.1 (A/B) 0.5 ± 0.1 (A/C)	**29 ± 7**	0.21 ± 0.05	0.01–100
**Desloratadine (A)** *w/NADPH (B)* *w/cofactors (C)*	150 ± 17 130 ± 27 146 ± 17	1.2 ± 0.3 (A/B) 1.0 ± 0.2 (A/C)	**23 ± 5**	128 ± 9 *99 ± 12* *178 ± 20*	1.3 ± 0.2 (A/B) 0.7 ± 0.1 (A/C)	**20 ± 4**	0.15 ± 0.03	0.01–100
**Disulfiram (A)** *w/NADPH (B)* *w/cofactors (C)*	32 ± 8 34 ± 11 -	0.9 ± 0.4 (A/B) n/a (A/C)	**29 ± 15**	76 ± 15 *133 ± 37* -	0.6 ± 0.2 (A/B) n/a (A/C)	**69 ± 35**	0.91 ± 0.42	0.01–25
**Esomeprazole (A)** *w/NADPH (B)* *w/cofactors (C)*	8.4 ± 1.0 3.6 ± 1.0 5.1 ± 0.9	2.3 ± 0.7 (A/B) 1.7 ± 0.4 (A/C)	**3.0 ± 0.8**	**155 ± 11** *133 ± 14* *202 ± 22*	1.2 ± 0.2 (A/B) 0.8 ± 0.1 (A/C)	**55 ± 13**	0.36 ± 0.08	0.01–100
**Flecainide (A)** *w/NADPH (B)* *w/cofactors (C)*	228 ± 34 258 ± 26 230 ± 41	0.9 ± 0.2 (A/B) 1.0 ± 0.2 (A/C)	**82 ± 14**	-	-	n/a	0.36 ± 0.03	0.01–150
**Orphenadrine (A)** *w/NADPH (B)* *w/cofactors (C)*	225 ± 55 192 ± 42 201 ± 26	1.2 ± 0.4 (A/B) 1.1 ± 0.3 (A/C)	**62 ± 19**	525 ± 76 *483 ± 107* *840 ± 177*	1.1 ± 0.3 (A/B) 0.6 ± 0.2 (A/C)	**144 ± 35**	0.27 ± 0.05	0.01–500
**Prazosin (A)** *w/NADPH (B)* *w/cofactors (C)*	21 ± 2 26 ± 4 36 ± 6	0.8 ± 0.1 (A/B) 0.6 ± 0.1 (A/C)	*	-	-	n/a	*	0.01–75
**Quetiapine (A)** *w/NADPH (B)* *w/cofactors (C)*	52 ± 7 63 ± 10 66 ± 6	0.8 ± 0.2 (A/B) 0.8 ± 0.1 (A/C)	**12 ± 3**	198 ± 21 *204 ± 19* *214 ± 26*	1.0 ± 0.1 (A/B) 0.9 ± 0.1 (A/C)	**46 ± 12**	0.24 ± 0.06	0.01–100

* The unbound fraction of prazosin could not be determined due to rapid precipitation preventing HPLC, analysis.

To evaluate the propensity for time-dependent inhibition (i.e., formation of reactive or inhibitory metabolites via P450), the test substances (each separately) were preincubated together with the P450 cofactor, NADPH, and RT-S9 prior to initiation of the EROD reaction (Figure 1, Series B). As a result, a significant IC_50_ shift (2.3 ± 0.7) was observed for esomeprazole (Figure 1C), indicating that it is a time-dependent inhibitor of EROD activity in rainbow trout *in vitro* (Table 3). None of the other test substances triggered a similarly significant fold change in their IC_50_ values determined with and without NADPH preincubation.

To evaluate the biological importance of the P450 inhibition, the test substances (each separately) were also preincubated with all cofactors, including NADPH and those of the phase II transferase enzymes (Series C), like in the liver cells. As a result, the inhibitory impact of disulfiram attenuated, suggesting that on a cellular level, its EROD inhibition is likely insignificant (Figure 1B). However, the IC_50_ shift observed for esomeprazole between Series A and B persisted even if esomeprazole was preincubated with all cofactors (Series C) and yielded an IC_50_ shift of 1.7 ± 0.4 (Figure 1C). This suggests that esomeprazole could cause time-dependent inhibition on a cellular level as well, which may be biologically relevant in rainbow trout *in vivo*. For all other substances, no significant changes were observed between IC_50_ values determined with and without preincubation with all cofactors (Table 3), indicating that their possible depletion (metabolic clearance) in RT-S9 is not fast enough to overrule their inhibitory impacts toward the EROD activity. It should also be noted that the apparent increase in the EROD activity observed at the highest felbinac concentrations (Figure 1A, all Series A-C) is likely resulting from an experimental artefact, rather than induction of this enzyme activity, which cannot take place in the subcellular S9 fractions not containing the P450 genes.

### 3.2 Inhibition of BFCOD activity by the test substances in rainbow trout liver S9 fractions

**FIGURE 2:**
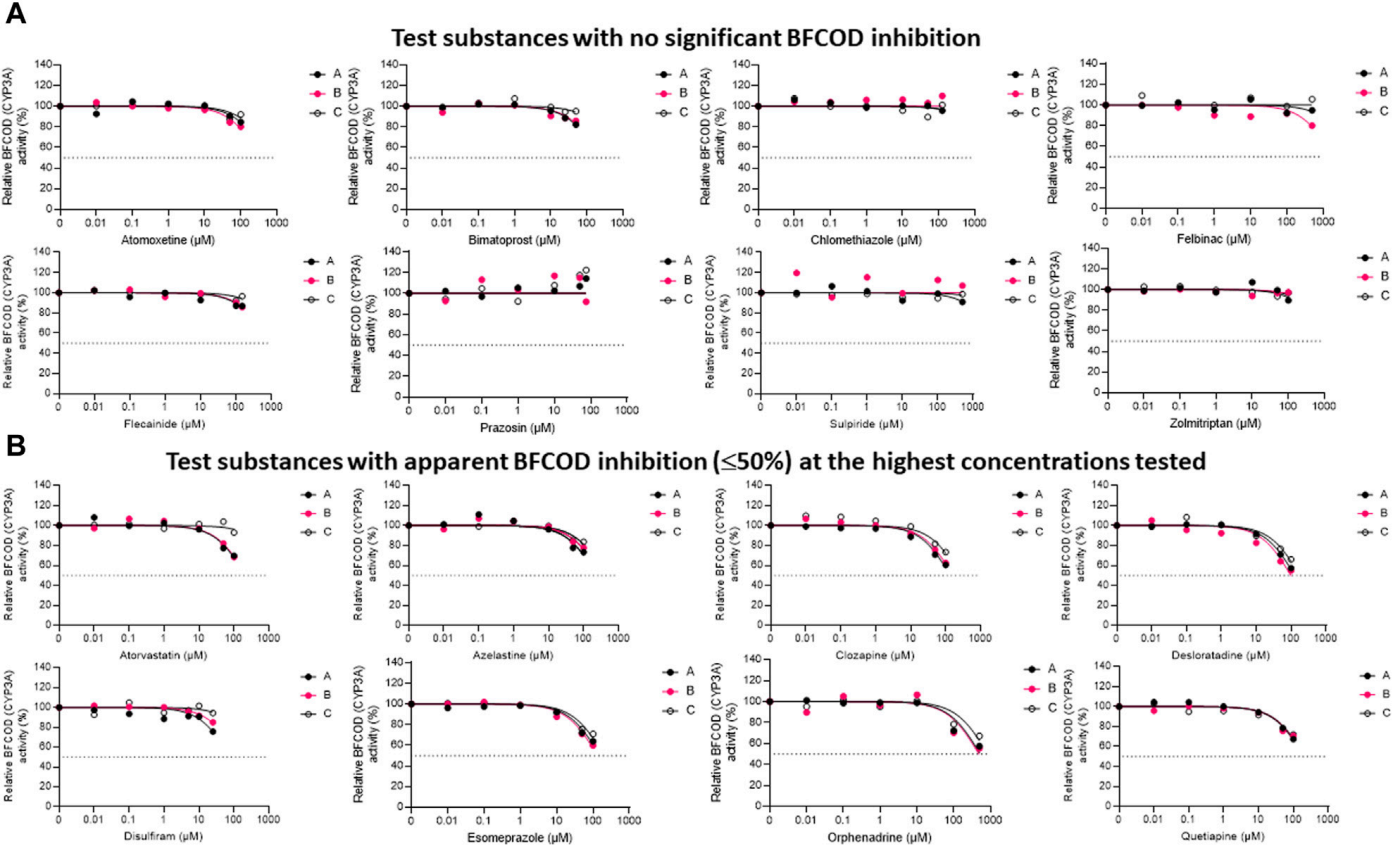
The nonlinear regression analyses of the residual BFCOD activities at different concentrations of the test pharmaceuticals in RT-S9, when the test substance and RT-S9 (1 mg/mL) were preincubated for 30 min without any cofactors (Series A), with 2 mM NADPH (Series B), or with all cofactors, including 2 mM NADPH, 1 mM UDPGA, 0.05 mM PAPS, and 2.5 mM glutathione (Series C), prior to initiation of the BFCOD marker reaction (20 min). On the basis of BFCOD inhibition, the test substances were grouped between **(A)** those that did not significantly inhibit BCFOD activity within the tested concentration range and **(B)** those that had apparent BFCOD inhibition of ≤50% at the highest test concentration. Each datapoint in each of the Series A-C represents an average of n = 2 replicate incubations.

The inhibitory impacts of the test substances toward rainbow trout CYP3A-like activity, assessed through nonlinear regression analyses of the residual BFCOD activities in RT-S9 assays at different pharmaceutical concentrations, are illustrated in Figure 2. On the basis of these results (Series B), half of the test substances (atomoxetine, bimatoprost, clomethiazole, felbinac, flecainide, prazosin, sulpiride, zolmitriptan) did not significantly inhibit the BFCOD activity in RT-S9 within the concentration range tested (Figure 2A). None of these substances are inhibitors of human CYP3A4/5 either, although bimatoprost, clomethiazole, and prazosin are substrates of human CYP3A4/5. Instead, the other half of the test substances (atorvastatin, azelastine, clozapine, desloratadine, disulfiram, esomeprazole, orphenadrine, quetiapine) triggered weak, apparent inhibition (≤50%) of the BFCOD activity in RT-S9 at the highest concentrations tested (Figure 2B), yielding extrapolated nominal IC_50_ values typically >100 µM (Table 3), apart from sulpiride (76 ± 15 µM). Notably, four of these substances (atorvastatin, azelastine, esomeprazole, orphenadrine) are also human CYP3A4/5 inhibitors and two (clozapine, quetiapine) are human CYP3A4/5 substrates (Table 1), indicating that qualitative correlation between the substrate selectivities of rainbow trout and human CYP3A homologues is somewhat better than that of CYP1A homologue. Among the test substances that inhibited rainbow trout CYP3A-like activity (Figure 2B), only desloratadine and disulfiram do not have known interactions with human CYP3A4/5.

To evaluate the propensity for time-dependent inhibition, the test substances (each separately) were preincubated together with the P450 cofactor, NADPH, and RT-S9 prior to initiation of the BFCOD reaction (Figure 2, Series B). As a result, none of the test substances were identified as time-dependent inhibitors of CYP3A-like activity in rainbow trout *in vitro*, based on the IC_50_ shift (i.e., no significant changes between Series A and B).

To evaluate the biological importance of the P450 inhibition, the test substances (each separately) were also preincubated with all cofactors, including NADPH and those of the phase II transferase enzymes (Figure 2, Series C). As a result, the inhibitory impacts of disulfiram (like in the case of EROD inhibition) and atorvastatin attenuated, indicating that their own metabolic clearance is fast enough to overrule their inhibitory impacts. For all other substances, no significant changes (shifts) were observed in the IC_50_ concentrations between Series A and C.

### 3.3 Nonspecific binding of the test substances to rainbow trout microsomal proteins

To further evaluate the biological importance of the observed EROD and BFCOD inhibition, we additionally determined the nonspecific binding of the test substances to the RT-S9 fractions using equilibrium dialysis. The unbound IC_50_ values of the parent pharmaceuticals (Series A), calculated based on their unbound fractions in RT-S9 (f_U,RT-S9_), are given in Table 3. Based on their inhibitory concentrations toward human CYPs, pharmaceuticals are typically classified between potent or strong inhibitors (IC_50_ < 1 µM), moderate inhibitors (1 µM < IC_50_ < 10 µM), weak inhibitors (IC_50_ > 10 µM), or noninhibitors (no significant P450 interaction). According to this classification, and accounting for the nonspecific binding of the test substances to RT-S9, azelastine and esomeprazole can be classified as fairly strong to moderate inhibitors of EROD activity in rainbow trout *in vitro*, with unbound IC_50_ values of 3.8 ± 0.5 µM and 3.0 ± 0.8 µM, respectively. Moreover, as many as six of the test substances are moderate to weak inhibitors of the EROD activity (atomoxetine, atorvastatin, clozapine, desloratadine, disulfiram, quetiapine), and clozapine and desloratadine additionally of the BFCOD activity, each with unbound IC_50_ between 10 and 30 µM in RT-S9. Instead, all other substances can be classified as weak or noninhibitors of rainbow trout CYP1A and CYP3A-like activities, even if their nonspecific binding to RT-S9 is accounted for.

**FIGURE 3:**
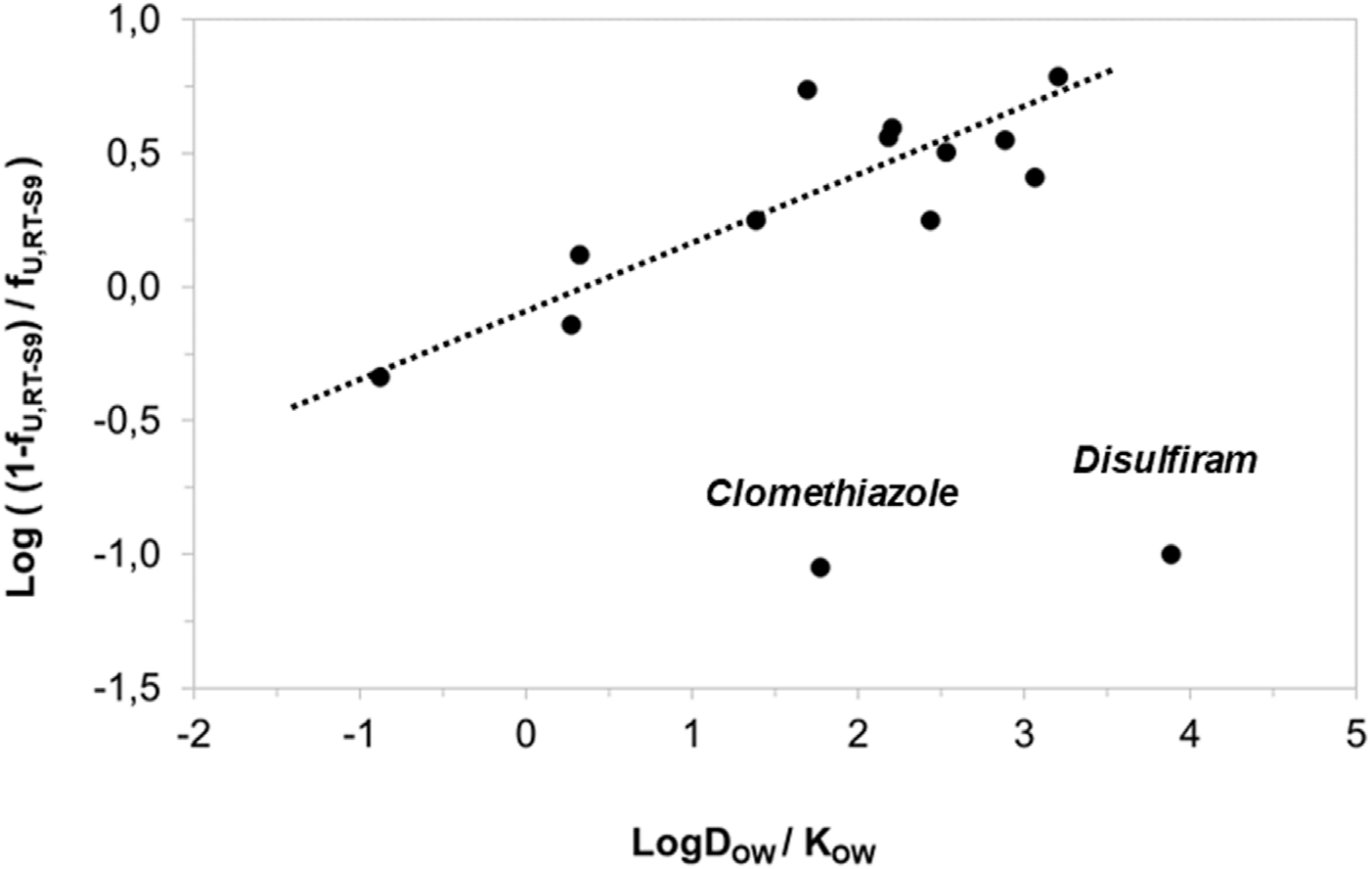
Correlation of the test substances’ nonspecific binding (unbound fractions) in RT-S9 with their water-octanol distribution coefficients (LogD_OW_ at the pH 7.8, for basic and acidic substances) or partitioning coefficients (LogK_OW_ for neutral substances). The unbound fractions (f_U,RT-S9_) represent the experimental values (n = 3 replicate assays with each test substance at 10 µM concentration).The LogD_OW_ and LogK_OW_ values are from Chemaxon and PubChem databases, respectively. The regression line represents the linear correlation (*R* ^2^ = 0.7135) with 95% confidence interval.

Besides IC_50_ correction, we also assessed the correlation of the nonspecific binding of the test substances to RT-S9 with their water-octanol distribution coefficients (logD_OW_) at pH 7.8. The correlation was established similar to the previous work (Austin et al., 2002), using log_10_ [(1-f_U,RT-S9_)/f_U,RT-S9_], which is analogous to the equilibrium constant of Eq. 3, and representative of the linear free energy relationships. As a result, strong correlation (R_2_ = 0.7135) between the log_10_ of nonspecific binding [(1-f_U,RT-S9_)/f_U,RT-S9_] and water-octanol distribution (D_OW_) of the test substances was observed (Figure 3). However, similar to rat liver microsomes (Austin et al., 2002), the substances which had negligible nonspecific binding to RT-S9 (f_U,RT-S9_>0.9), did not comply with the established correlation. These substances included clomethiazole and disulfiram, both neutral at the pH 7.8, which likely explains their negligible nonspecific binding to RT-S9 and is well in line with the previous observations made using rat liver microsomes (Austin et al., 2002).

## 4 Discussion

In the present study, we assessed the *in vitro* EROD and BFCOD inhibition in RT-S9 by a range of pharmaceuticals from different therapeutic classes (Table 1), giving priority to substances lacking or having only limited environmental fate data so far (Cannata et al., 2024). The EROD reaction is primarily catalyzed by CYP1A-like activity in fish and human alike. In rainbow trout, the CYP1A1 isoform is abundantly expressed in the liver as well as most other tissues (Burkina et al., 2021) and it is one of the most studied P450 isoforms, because the EROD activity is reportedly induced by a multitude of chemicals, including pharmaceuticals, in fish *in vivo* (Whyte et al., 2000). Consequently, the BFCOD reaction is primarily catalyzed by CYP3A-like activity in fish. Similar to CYP1A1, rainbow trout CYP3A27 is abundantly expressed in the liver, displaying 54.9% sequence homology to human CYP3A4 (Lee et al., 1998).

The first objective of this study was to evaluate the qualitative correlation between the substrate selectivities between human and rainbow trout CYP1A and CYP3A homologs. Human CYP1A favors planar, relatively small aromatic molecules (Dong et al., 2012; Dai et al., 2024). None of the pharmaceuticals assessed in this study are categorically strong inhibitors (IC_50_ < 1 µM) of human CYP1A activity, but weak or predicted CYP1A inhibition is associated with orphenadrine and desloratadine, respectively (Table 1). In addition, four other test substances (azelastine, clozapine, flecainide, zolmitriptan) are substrates of human CYP1A. Instead, the human CYP3A4/5 homologue has a fairly large active site (ca. 1000–2000 Å^3^) and consequently, it catalyzes nearly 50% of the biotransformation reactions of clinically used pharmaceuticals (Wienkers and Heath, 2005; Zanger et al., 2008; Dong et al., 2012), including nine of the pharmaceuticals screened in the present study (atorvastatin, azelastine, bimatoprost, clomethiazole, clozapine, esomeprazole, orphenadrine, prazosin, quetiapine). In addition, four of the test substances are known inhibitors of human CYP3A4/5 (atorvastatine, azelastine, esomeprazole, orphenadrine), whereas the remaining substances (atomoxetine, desloratadine, disulfiram, felbinac, flecainide, sulpiride, zolmitriptan) do not have known interactions with human CYP3A4/5 (Table 1). Nevertheless, most of the pharmaceuticals assessed in the present study resulted in a fairly broad inhibition of both EROD (CYP1A-like) and BFCOD (CYP3A-like) activity in RT-S9. Three substances (atomoxetine, flecainide, prazosin) were selective inhibitors of EROD, but not BFCOD activity, and all other test substances had interactions with both marker reactions in RT-S9. However, the inhibitory impact of disulfiram, toward both EROD and BFCOD, attenuated as the result of its intrinsic clearance in the RT-S9. Similar attenuation was also observed in the inhibitory impact of atorvastatin toward BFCOD, but not EROD, activity. For all other test substances, the inhibitory impact persisted despite of their preincubation with RT-S9 prior to initiation of the marker reaction. It is also noteworthy, that only five of the test substances (bimatoprost, clomethiazole, felbinac, sulpiride, zolmitriptan) did not inhibit either EROD or BFCOD in RT-S9, even if majority of the test substances are noninhibitors of the corresponding human CYP1A or CYP3A homologues. Thus, it may be concluded that definitive read-across from human P450 interactions is not necessarily feasible, but the substrate specificities of rainbow trout CYP1A and CYP3A are likely much broader than those of the human homologs. Consequently, it may be hypothesized that P450 inhibition in rainbow trout can play a role in the combined effects of environmental chemicals and their mixtures. Correction of the *in vitro* endpoints for nonspecific binding of the test substances is however necessary with a view to in vitro-in vivo extrapolation.

Previous work has demonstrated that pharmaceuticals’ microsomal binding can be qualitatively predicted from their physicochemical characteristics: strong nonspecific binding to both human and rat liver microsomes has been associated especially with lipophilic and basic substances, and typically to a lesser degree with acidic or neutral pharmaceuticals (McLure et al., 2000; Austin et al., 2002). Advanced models for prediction of the nonspecific binding in fish tissues and tissue preparations have also been established in recent years to help assess chemicals’ bioaccumulation based on *in vitro* endpoints (Laue et al., 2020; Saunders and Nichols, 2023). The nonspecific binding of the pharmaceuticals assessed in this study was generally in good agreement with previous literature manifesting similar correlation between RT-S9 binding and physicochemical characteristics, as has been previously reported for pharmaceuticals’ binding to human and rat liver microsomes (McLure et al., 2000; Austin et al., 2002). In mammals, binding to hepatic tissue preparations, such as liver microsomes, can even serve as a surrogate for their predicted tissue binding (Ryu et al., 2020; Hsu et al., 2021). Consequently, the nonspecific binding data reported herein can help predict the test substances’ binding to fish tissues, and consequently aid physiologically based pharmacokinetic modelling and associated bioaccumulation risk assessment.

The second objective of this study was to evaluate the biological importance of the P450 inhibition in rainbow trout by pharmaceuticals considering their environmental occurrence. Accounting for the nonspecific binding to RT-S9, the inhibitory impacts of the test pharmaceuticals toward the BFCOD activity were weak (unbound IC_50_ > 10 µM) or very weak (unbound IC_50_ > 100 µM), whereas the EROD inhibition was categorically stronger resulting in lower IC_50_ concentrations. The strongest EROD inhibition was associated with azelastine (unbound IC_50_ = 3.8 ± 0.5 µM) and esomeprazole (unbound IC_50_ = 3.0 ± 0.8 µM), followed by atorvastatin and clozapine (unbound IC_50_ ca. 10 μM each). Overall, these threshold concentrations are approximately at the same level or slightly higher than those generally reported for human P450s. From the perspective of environmental risk assessment, however, the measured IC_50_ concentrations in RT-S9, even for EROD activity, are many orders of magnitude higher than the average environmental concentrations of pharmaceuticals in surface waters. Upon bioaccumulation, the pharmaceuticals’ tissue concentrations can theoretically increase thousand folds closer to the threshold concentrations triggering EROD inhibition. Nevertheless, it may be concluded unlikely that the threshold concentrations would exceed in fish liver *in vivo*. Namely, both CYP1A-like (EROD) and CYP3A-like (BFCOD) activities are also inducible in fish by a range of chemicals (Whyte et al., 2000; Oziolor et al., 2017), which can overcome the inhibitory impacts in wild fish that are continuously exposed to chemical contaminants. However, key uncertainties relate to mixture effects targeting the fish P450 system. Namely, previous studies have established evidence of synergistic P450 inhibition in fish *in vitro* and *in vivo* (Hasselberg et al., 2005; Pihlaja et al., 2022), highlighting the complexity of the combined impacts of pharmaceuticals on fish and challenging the currently available mixture toxicity models based on concentration addition assumption. In this regard, the *in vitro* data produced herein can shed light on the mechanistic basis of the synergistic effects possibly observed in fish *in vivo* even at low environmental concentrations.

Finally, the third objective of this study was to assess the propensity for irreversible P450 inhibition (often resulting from the formation of reactive, and thus toxic metabolites) by these test pharmaceuticals in rainbow trout via the IC_50_ shift assay. Among the test substances, esomeprazole was the only time-dependent inhibitor of EROD, but not BFCOD, activity, suggesting that its own biotransformation in rainbow trout liver may produce metabolites that are reactive and can potentially trigger irreversible inhibition of the CYP1A-like activity. In human use, esomeprazole is a prodrug, i.e., its mode of action is based on a pharmacologically active metabolite produced in the human body. More precisely, in human gastric parietal cells, esomeprazole, undergoes activation to its reactive metabolite sulfenamide, which covalently binds to an enzyme, irreversibly blocking the proton pump system. Although there is no direct evidence of the involvement of P450s in the formation of the sulfenamide metabolite, the required proton transfer reaction can theoretically be catalyzed by the oxido-reductive P450 system. Thus, it may be hypothesized that the time-dependent EROD inhibition by esomeprazole in RT-S9 could result from the formation of reactive metabolites and warrant for further research regarding its interlinkages with possible hepatotoxic effects on fish. It should be noted however, that prodrugs like esomeprazole are not extensively excreted in their parent form in humans, and thus their environmental concentrations are likely very low, which consequently lowers the risk of esomeprazole exposure in wild fish.

Altogether, the *in vitro* data produced in the present study will contribute to environmental risk assessment of these particular pharmaceuticals, for which the environmental effects and fate data is currently very limited (Cannata et al., 2024). Besides esomeprazole, none of the other test substances were time-dependent inhibitors of neither EROD nor BFCOD activity in RT-S9. Thus, it may be concluded that these particular test substances do not pose a major risk for irreversible P450 inhibition in rainbow trout *in vivo* and that their inhibitory threshold concentrations are categorically well above the average environmental concentrations of pharmaceuticals. However, further studies are necessary to thoroughly evaluate their fate and effects in fish tissues, such as intrinsic clearance rates and plasma binding.

## Data Availability

The original contributions presented in the study are included in the article/Supplementary Material, further inquiries can be directed to the corresponding author.

## Abbreviations

API, active pharmaceutical ingredient; BFC(OD), 7-benzyloxy-4-trifluoromethylcoumarin (O-debenzylation); CYP, cytochrome P450; ER (OD), ethoxyresorufin (O-deethylation); GST, glutathione-S-transferases; IC50, half-maximal inhibitory concentration; LogDOW, octanol-water distribution coefficient; LogKOW, octanol-water partition coefficient; NADPH, nicotinamide adenine dinucleotide phosphate; OECD, Organisation for Economic Co-operation and Development; PAPS, 3′-Phosphoadenosine-5′-phosphosulfate; RED, rapid equilibrium dialysis; RTL-S9, rainbow trout liver S9 subcellular fraction; SULT, sulfotransferases; UDPGA, uridine diphosphate glucuronic acid; UGT, uridine 5′-diphospho-glucuronosyltransferase.

## References

[B1] aus der Beek T. Weber F. A. Bergmann A. Hickmann S. Ebert I. Hein A. (2016). Pharmaceuticals in the environment - global occurrences and perspectives. Environ. Toxicol. Chem. 35, 823–835. 10.1002/etc.3339 26666847

[B2] Austin R. P. Barton P. Cockroft S. L. Wenlock M. C. Riley R. J. (2002). The influence of nonspecific microsomal binding on apparent intrinsic clearance, and its prediction from physicochemical properties. Drug Metab. Dispos. 30 (12), 1497–1503. 10.1124/dmd.30.12.1497 12433825

[B3] Berry L. M. Zhao Z. (2008). An examination of IC50 and IC50-shift experiments in assessing time-dependent inhibition of CYP3A4, CYP2D6 and CYP2C9 in human liver microsomes. Drug Metab. Lett. 2, 51–59. 10.2174/187231208783478407 19356071

[B4] Brodin T. Fick J. Jonsson M. Klaminder J. (2013). Dilute concentrations of a psychiatric drug alter behavior of fish from natural populations. Science 339 (6121), 814–815. 10.1126/science.1226850 23413353

[B5] Brown J. N. Paxéus N. Förlin L. Larsson D. G. J. (2007). Variations in bioconcentration of human pharmaceuticals from sewage effluents into fish blood plasma. Environ. Toxicol. Pharmacol. 24 (3), 267–274. 10.1016/j.etap.2007.06.005 21783821

[B6] Burkina V. Zamaratskaia G. Sakalli S. Giang P. T. Zlabek V. Krøyer Rasmussen M. (2021). Tissue-specific expression and activity of cytochrome P450 1A and 3A in rainbow trout (*Oncorhynchus mykiss*). Toxicol. Lett. 341, 1–10. 10.1016/j.toxlet.2021.01.011 33429014

[B7] Burkina V. Zlabek V. Zamaratskaia G. (2013). Clotrimazole, but not dexamethasone, is a potent *in vitro* inhibitor of cytochrome P450 isoforms CYP1A and CYP3A in rainbow trout. Chemosphere 92, 1099–1104. 10.1016/j.chemosphere.2013.01.050 23466084

[B8] Cannata C. Backhaus T. Bramke I. Caraman M. Lombardo A. Whomsley R. (2024). Prioritisation of data-poor pharmaceuticals for empirical testing and environmental risk assessment. Environ. Int. 183, 108379. (10 pp). 10.1016/j.envint.2023.108379 38154319

[B9] Cerveny D. Grabic R. Grabicová K. Randák T. Larsson D. G. J. Johnson A. J. (2021). Neuroactive drugs and other pharmaceuticals found in blood plasma of wild European fish. Environ. Int. 146, 106188. 10.1016/j.envint.2020.106188 33096467

[B10] Christen V. Oggier D. M. Fent K. (2009). A microtiter-plate-based cytochrome P450 3A activity assay in fish cell lines. Environ. Toxicol. Chem. 28, 2632–2638. 10.1897/08-483.1 19245271

[B11] Cohen L. H. van Leeuwen R. E. W. van Thiel G. C. F. van Pelt J. F. Yap S. H. (2000). Equally potent inhibitors of cholesterol synthesis in human hepatocytes have distinguishable effects on different cytochrome P450 enzymes. Biopharm. Drug Dispos. 21, 353–364. 10.1002/bdd.249 11523064

[B12] Conard G. J. Ober R. E. (1984). Metabolism of flecainide. Am. J. Cardiol. 53 (5), 41B-51B–51B. 10.1016/0002-9149(84)90501-0 6364769

[B14] Dai Z. Wu Y. Xiong Y. Wu J. Wang M. Sun X. (2024). CYP1A inhibitors: recent progress, current challenges, and future perspectives. Med. Res. Rev. 44 (1), 169–234. 10.1002/med.21982 37337403

[B15] Dong D. Wu B. Chow D. Hu M. (2012). Substrate selectivity of drug-metabolizing cytochrome P450s predicted from crystal structures and *in silico* modeling. Drug Metab. Dispos. 44, 192–208. 10.3109/03602532.2011.645580 22251142

[B16] Dragovic S. Gunness P. Ingelman-Sundberg M. Vermeulen N. P. Commandeur J. N. (2013). Characterization of human cytochrome P450s involved in the bioactivation of clozapine. Drug Metab. Dispos. 41 (3), 651–658. 10.1124/dmd.112.050484 23297297

[B17] Eide M. Zhang X. Karlsen O. A. Goldstone J. V. Stegeman J. Jonassen I. (2021). The chemical defensome of five model teleost fish. Sci. Rep. 11, 10546–10613. 10.1038/s41598-021-89948-0 34006915 PMC8131381

[B18] Fontana E. Dansette P. M. Poli S. M. (2005). Cytochrome p450 enzymes mechanism based inhibitors: common sub-structures and reactivity. Curr. Drug Metab. 6 (5), 413–454. 10.2174/138920005774330639 16248836

[B19] Franco M. E. Schönenberger R. Hollender J. Schirmer K. (2024). Organ-specific biotransformation in salmonids: insight into intrinsic enzyme activity and biotransformation of three micropollutants. Sci. Total Environ. 925 (11), 171769. 10.1016/j.scitotenv.2024.171769 38499104

[B20] Gómez-Regalado M. D. C. Martín J. Santos J. L. Aparicio I. Alonso E. Zafra-Gómez A. (2023). Bioaccumulation/bioconcentration of pharmaceutical active compounds in aquatic organisms: assessment and factors database. Sci. Total Environ. 861, 160638. 10.1016/j.scitotenv.2022.160638 36473663

[B21] Grabicova K. Grabic R. Fedorova G. Fick J. Cerveny D. Kolarova J. (2017). Bioaccumulation of psychoactive pharmaceuticals in fish in an effluent dominated stream. Water Res. 124, 654–662. 10.1016/j.watres.2017.08.018 28825984

[B22] Guengerich F. P. (2006). Cytochrome P450s and other enzymes in drug metabolism and toxicity. AAPS J. 8, E101–E111. 10.1208/aapsj080112 16584116 PMC2751428

[B23] Gunnarsson L. Snape J. R. Verbruggen B. Owen S. F. Kristiansson E. Margiotta-Casaluci L. (2019). Pharmacology beyond the patient – the environmental risks of human drugs. Environ. Int. 129, 320–332. 10.1016/j.envint.2019.04.075 31150974

[B24] Guo Z. Raeissi S. White R. B. Stevens J. C. (1997). Orphenadrine and methimazole inhibit multiple cytochrome P450 enzymes in human liver microsomes. Drug Metab. Dispos. 25 (3), 390–393. 9172960

[B25] Hasselberg L. Grøsvik B. E. Goksøyr A. Celander M. C. 2005. Interactions between xenoestrogens and ketoconazole on hepatic CYP1A and CYP3A, in juvenile Atlantic cod (*Gadus morhua*). Comp. Hepatol 4(1), 2pp), 10.1186/1476-5926-4-2 15701172 PMC549046

[B26] Hegelund T. Ottosson K. Rådinger M. Tomberg P. Celander M. C. (2004). Effects of the antifungal imidazole ketoconazole on CYP1A and CYP3A in rainbow trout and killifish. Environ. Toxicol. Chem. 23, 1326–1334. 10.1897/03-155 15180387

[B27] Hsu F. Chen Y. C. Broccatelli F. (2021). Evaluation of tissue binding in three tissues across five species and prediction of volume of distribution from plasma protein and tissue binding with an existing model. Drug Metab. Dispos. 49, 330–336. 10.1124/dmd.120.000337 33531412

[B28] Jobling S. Williams R. Johnson A. Taylor A. Gross-Sorokin M. Nolan M. (2006). Predicted exposures to steroid estrogens in U.K. rivers correlate with widespread sexual disruption in wild fish populations. Environ. Health Perspect. 114 (Suppl. 1), 32–39. 10.1289/ehp.8050 16818244 PMC1874167

[B29] Jönsson M. Abrahamson A. Brunström B. Brandt I. (2006). Cytochrome P4501A induction in rainbow trout gills and liver following exposure to waterborne indigo, benzo[a]pyrene and 3,3',4,4',5-pentachlorobiphenyl. Aquat. Toxicol. 79, 226–232. 10.1016/j.aquatox.2006.06.006 16872689

[B30] Laue H. Hostettler L. Badertscher R. P. Jenner K. J. Sanders G. Arnot J. A. (2020). Examining uncertainty in *in vitro*–*in vivo* extrapolation applied in fish bioconcentration models. Environ. Sci. Technol. 54 (15), 9483–9494. 10.1021/acs.est.0c01492 32633948

[B31] Lee D. Choi K. (2019). Comparison of regulatory frameworks of environmental risk assessments for human pharmaceuticals in EU, USA, and Canada. Sci. Total Environ. 671, 1026–1035. 10.1016/j.scitotenv.2019.03.372

[B32] Lee S. J. Wang-Buhler J. L. Cok I. Yu T. S. Yang Y. H. Miranda C. L. (1998). Cloning, sequencing, and tissue expression of CYP3A27, a new member of the CYP3A subfamily from embryonic and adult rainbow trout livers. Arch. Biochem. Biophys. 360, 53–61. 10.1006/abbi.1998.0943 9826429

[B33] Li A. P. (2002). A review of the common properties of drugs with idiosyncratic hepatotoxicity and the "multiple determinant hypothesis" for the manifestation of idiosyncratic drug toxicity. Chem. Biol. Interact. 142 (1-2), 7–23. 10.1016/s0009-2797(02)00051-0 12399152

[B34] McLure J. A. Miners J. O. Birkett D. J. (2000). Nonspecific binding of drugs to human liver microsomes. Br. J. Clin. Pharmacol. 49, 453–461. 10.1046/j.1365-2125.2000.00193.x 10792203 PMC2014956

[B35] Miranda C. L. Henderson M. C. Buhler D. R. (1998). Evaluation of chemicals as inhibitors of trout cytochrome P450s. Toxicol. Appl. Pharmacol. 148, 237–244. 10.1006/taap.1997.8341 9473531

[B36] Muir D. Simmons D. Wang X. Peart T. Villella M. Miller J. (2017). Bioaccumulation of pharmaceuticals and personal care product chemicals in fish exposed to wastewater effluent in an urban wetland. Sci. Rep. 7, 16999. 10.1038/s41598-017-15462-x 29208974 PMC5717258

[B37] Niwa T. Inoue S. Shiraga T. Takagi A. (2005). No inhibition of cytochrome P450 activities in human liver microsomes by sulpiride, an antipsychotic drug. Biol. Pharm. Bull. 28, 188–191. 10.1248/bpb.28.188 15635191

[B38] Obach R. S. Walsky R. L. Venkatakrishnan K. (2007). Mechanism-based inactivation of human cytochrome p450 enzymes and the prediction of drug-drug interactions. Drug Metab. Dispos. 35, 246–255. 10.1124/dmd.106.012633 17093004

[B39] Oziolor E. M. Carey A. N. Matson C. W. (2017). A non-destructive BFCOD assay for *in vivo* measurement of cytochrome P450 3A (CYP3A) enzyme activity in fish embryos and larvae. Ecotoxicology 26 (6), 809–819. 10.1007/s10646-017-1812-5 28589335

[B40] Pihlaja T. Niemissalo S. Sikanen T. (2022). Cytochrome P450 inhibition by antimicrobials and their mixtures in rainbow trout liver microsomes *in vitro* . Environ. Toxicol. Chem. 41, 663–676. 10.1002/etc.5160 34255900

[B42] Ryu S. Tess D. Chang G. Keefer C. Burchett W. Steeno G. S. (2020). Evaluation of fraction unbound across 7 tissues of 5 species. J. Pharm. Sci. 109 (2), 1178–1190. 10.1016/j.xphs.2019.10.060 31704191

[B43] Saunders L. J. Nichols J. W. (2023). Models used to predict chemical bioaccumulation in fish from *in vitro* biotransformation rates require accurate estimates of blood–water partitioning and chemical volume of distribution. Environ. Toxicol. Chem. 42 (1), 33–45. 10.1002/etc.5503 36282023 PMC10824487

[B44] Smith E. M. Iftikar F. I. Higgins S. Irshad A. Jandoc R. Lee M. (2012). *In vitro* inhibition of cytochrome P450–mediated reactions by gemfibrozil, erythromycin, ciprofloxacin and fluoxetine in fish liver microsomes. Aquat. Toxicol. 109, 259–266. 10.1016/j.aquatox.2011.08.022 22000335

[B46] Whyte J. J. Jung R. E. Schmitt C. J. Tillitt D. E. (2000). Ethoxyresorufin-O-deethylase (EROD) activity in fish as a biomarker of chemical exposure. Crit. Rev. Toxicol. 30, 347–570. 10.1080/10408440091159239 10955715

[B47] Wienkers C. L. Heath T. G. (2005). Predicting *in vivo* drug interactions from *in vitro* drug discovery data. Nat. Rev. Drug Discov. 4, 825–833. 10.1038/nrd1851 16224454

[B48] Wilkinson J. L. Boxall A. B. A. Kolpin D. W. Teta C. Lai R. W. S. Galbán-Malagón C. (2022). Pharmaceutical pollution of the world’s rivers. PNAS 19 (10), e2113947119. 10.1073/pnas.2113947119

[B49] Zanger U. M. Turpeinen M. Klein K. Schwab M. (2008). Functional pharmacogenetics/genomics of human cytochromes P450 involved in drug biotransformation. Anal. Bioanal. Chem. 392, 1093–1108. 10.1007/s00216-008-2291-6 18695978

