# Supplementary material for: Many human pharmaceuticals are weak inhibitors of the cytochrome P450 system in rainbow trout (Oncorhynchus mykiss) liver S9 fractions
Source: Front Toxicol. 2024 Jul 15;6:1406942. doi: 10.3389/ftox.2024.1406942 (PMC11284600; doi:10.3389/ftox.2024.1406942)
Supplement: Supplementary file 1 [file DataSheet1.docx]

Supplementary Material

Many Human Pharmaceuticals Are Weak Inhibitors of the Cytochrome P450 System in Rainbow Trout (*Oncorhynchus mykiss*) Liver S9 Fractions

Tea Pihlaja^1,2^, Timo Oksanen^1^, Netta Vinkvist^1^, Tiina Sikanen^1,2*^

^1^Faculty of Pharmacy, Drug Research Program, University of Helsinki, Helsinki, Finland

^2^Helsinki Institute for Sustainability Science, University of Helsinki, Helsinki, Finland

*** Correspondence:** Corresponding author: [tiina.sikanen@helsinki.fi](mailto:tiina.sikanen@helsinki.fi)

**Supplementary Table S1.** The enzyme activities of the commercial RT-S9 lot (#RTL-S9 200629-3) used in the study, as provided by the supplier (Primacyt Cell Culture Technology GmbH).

| **Marker activity (human enzyme selectivity)** | **Enzyme activity (nM/min) ***  Mean ± SD |
| --- | --- |
| Phenacetin-O-deethylase (CYP1A2) | 2.25±0.35 |
| Bupropion-hydroxylase (CYP2B6) | 0.24±0.04 |
| Diclofenac 4’-hydroxylase (CYP2C9) | 2.99±0.39 |
| Chlorzoxazone-6’-hydroxylase (CYP2E1) | 0.49 ± 0.25 |
| Midazolam 1’-hydroxylase (CYP3A4) | 1.78±0.91 |
| 7-Hydroxycoumarin glucuronidation (UGT) | 398.5±64.5 |
| 7-Hydroxycoumarin sulfonation (SULT) | 34.9±8.1 |
| ** The enzyme activities were determined at 1 mg/ml protein in 0.1 M phosphate buffer at 14 °C for 15 min (cytochrome P450, CYP) and for 30 min (UDP-glucuronosyl transferases, UGT; sulfotransferases, SULT) using a cocktail assay of known (human) marker activities of the respective enzymes.* | |

(a)
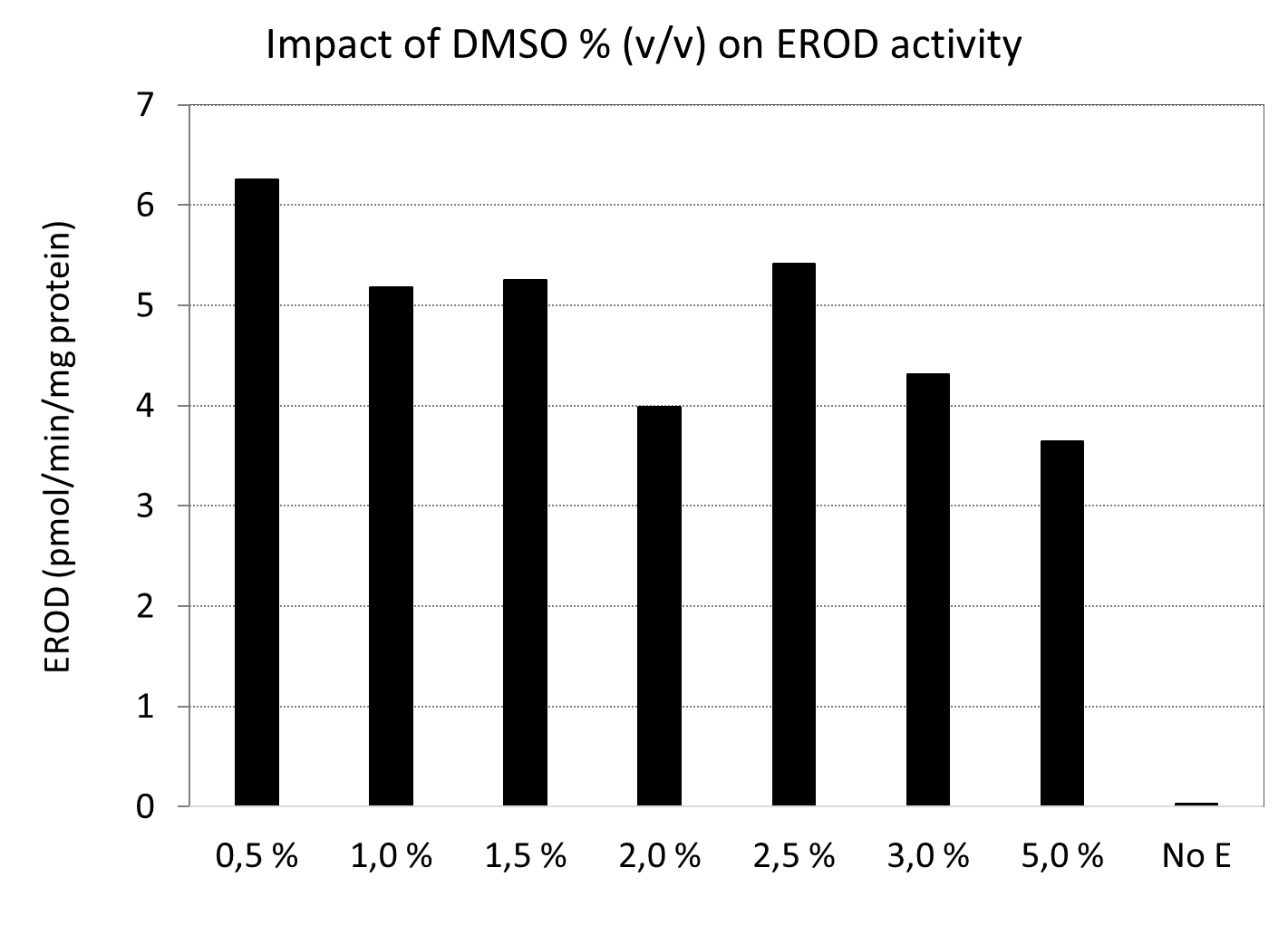
 (b)
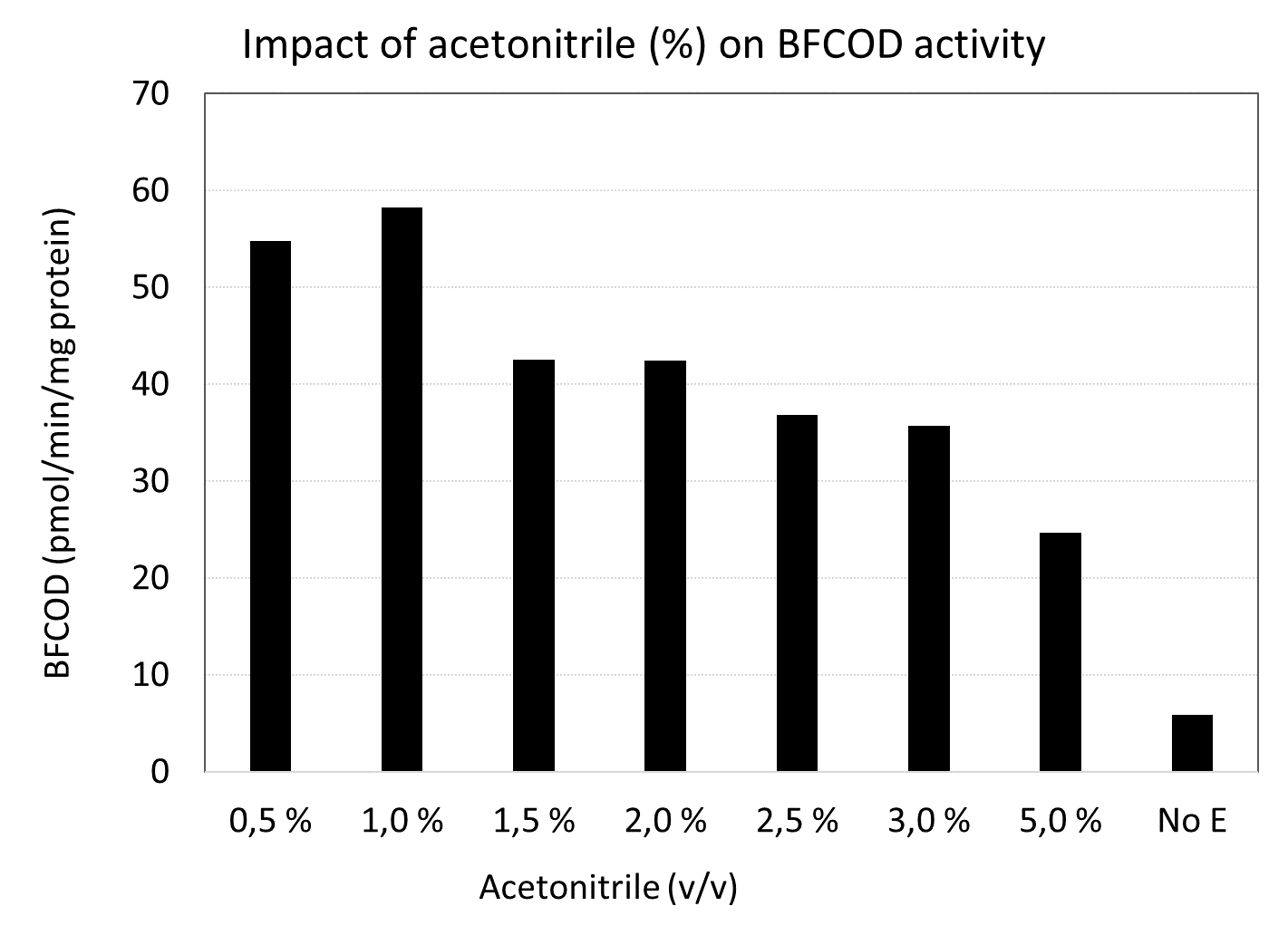


**Supplementary Figure S1.** The impacts of (a) residual dimethyl sulfoxide (DMSO) concentration on the basal EROD activity and (b) residual acetonitrile concentration on the basal BFCOD activity in rainbow trout liver microsomes. Each datapoint represents the average of n=2 replicate incubations. No E denotes negative control incubation conducted without the enzyme source. The incubations were carried out in potassium phosphate buffer (0.1M, pH 8.0) using 0.5 mg/mL total protein concentration and 1 mM (EROD) or 2 mM (BFCOD) concentration of NADPH. The reaction time was 10 min (EROD) or 20 min (BFCOD).

**Supplementary Table S2.** The analytical methods and instrumentation used for determination of the unbound fraction in RT-S9 (f_U,RT-S9_).

| **Compound** | **Atorvastatin** | | **Azelastine** | | **Clomethiazole** | | **Desloratadine** | | **Disulfiram** | | **Felbinac** | | **Sulpiride** | |
| --- | --- | --- | --- | --- | --- | --- | --- | --- | --- | --- | --- | --- | --- | --- |
| **Instrument** | Agilent 1200 Liquid chromatograph equipped with G1315C Diode array and G1321A Fluorescence detectors (desloratadine)  or Agilent 1260 Infinity II Liquid chromatograph equipped with G7115A Diode array and G7121B Fluorescence detectors (all other substances) | | | | | | | | | | | | | |
| **LC column** | Agilent InfinityLab Poroshell 120 EC-C18, 4.6 x 100 mm, 2.7 um + KrudKatcher, 2.0 µm (in-line filter) | | | | | | | | | | | | | |
| **Solvent gradients** | **Time**  **(min)** | **%B** | **Time**  **(min)** | **%B** | **Time**  **(min)** | **%B** | **Time**  **(min)** | **%B** | **Time**  **(min)** | **%B** | **Time**  **(min)** | **%B** | **Time**  **(min)** | **%B** |
|  | 0  5  10  12  13  13.5 | 10  60  80  100  100  10 | 0  6  6.5  9 | 60  100  60  60 | 0  6  6.5  9 | 60  100  60  60 | 0  1  4  4.05  4.5  4.6 | 10  10  25  90  90  15 | 0  6  6.5  9 | 60  100  60  60 | 0  5  10  12  13  13.5 | 10  60  80  100  100  10 | 0  6  6.5  9 | 60  100  60  60 |
| **t_R_ (min)** | 11.03 | | 7.58 | | 3.61 | | 2.80 | | 4.19 | | 3.48 | | 2.71 | |
| **V_injection_ (µL)** | 50 | | 50 | | 10 | | 5 | | 10 | | 10 | | 50 | |
| **Detection (nm)** | ex/em 260/392 | | ex/em 228/371 | | abs 254 | | ex/em 280/452 | | abs 260 | | ex/em 258/324 | | ex/em 228/357 | |
| **Linearity** | y=0.0080x-0.1974 | | y=0.0464x+0.0510 | | y=0.0140x-1.0995 | | y=0.0576x+0.0261 | | y=0.02155x+0.3648 | | y=0.0943x+1.5625 | | y=0.0073x-0.0730 | |
| **R^2^** | 0.9960 | | 0.9977 | | 0.9947 | | 0.9998 | | 0.9995 | | 0.9994 | | 0.9996 | |
| **Range (nM)** | 25-2000 | | 25-2000 | | 75-2000 | | 31.25-2000 | | 25-2000 | | 25-2000 | | 25-2000 | |
| **Compound** | **Atomoxetine** | | **Clozapine** | | **Esomeprazole** | | **Flecainide** | | **Orphenadrine** | | **Quetiapine** | | **Zolmitriptan** | |
| **Instrument** | Waters Acquity UPLC equipped with QDa Mass detector | | | | | | | | | | | | | |
| **LC column** | Phenomenex Luna Omega Polar C18 Column 1.6 µm particle size, 50 mm x 2.1 mm +0.2 µm online filter (Waters) | | | | | | | | | | | | | |
| **Solvent gradients** | **Time**  **(min)** | **%B** | **Time**  **(min)** | **%B** | **Time**  **(min)** | **%B** | **Time**  **(min)** | **%B** | **Time**  **(min)** | **%B** | **Time**  **(min)** | **%B** | **Time**  **(min)** | **%B** |
|  | 0.00  3.50  3.51  4.50 | 10  90  10  10 | 0.00  3.50  3.51  4.50 | 10  80  10  10 | 0.00  3.50  3.51  4.50 | 10  90  10  10 | 0.00  3.50  3.51  4.50 | 10  80  10  10 | 0.00  3.50  3.51  4.50 | 10  90  10  10 | 0.00  3.50  3.51  4.50 | 10  80  10  10 | 0.00  3.50  3.51  4.50 | 10  80  10  10 |
| **t_R_ (min)** | 1.77 | | 1.32 | | 1.21 | | 1.86 | | 1.79 | | 1.46 | | 0.82 | |
| **V_injection_ (µL)** | 2 | | 2 | | 2 | | 2 | | 2 | | 2 | | 2 | |
| **Detection (MS)** | SIR m/z 256.2 | | SIR m/z 327.2 | | SIR m/z 346.3 | | SIR m/z 415.2 | | SIR m/z 181.1/270.2 | | SIR m/z 384.3 | | SIR m/z 288.2 | |
| **Linearity** | y=4470x-1783 | | y=3460x-175 | | y=3502x-1930 | | y=7146x+1314 | | y=3179x-972 | | y=5127x-5982 | | y=2879x+470 | |
| **R^2^** | 0.9999 | | 0.9999 | | 0.9997 | | 0.9997 | | 0.9998 | | 0.9982 | | 0.9998 | |
| **Range (nM)** | 1-100 | | 5-100 | | 5-75 | | 1-100 | | 5-100 | | 5-75 | | 1-100 | |
